# Mechanistic investigation of a soybean protein isolate–soyasaponin–pectin composite system on soyasaponin bitterness in an acidic environment

**DOI:** 10.1016/j.fochx.2025.102500

**Published:** 2025-05-01

**Authors:** Lijie Zhu, Yingyan Li, He Li, Dayun Zhao, Xinqi Liu, Qian Shen, Qi Zhang, Qingyun Lv, Xiuying Liu, Wenping Ding

**Affiliations:** a Key Laboratory for Deep Processing of Major Grain and Oil, Ministry of Education; School of Food Science and Engineering, Wuhan Polytechnic University, Wuhan, Hubei 430028, China; b China Food Flavor and Nutrition Health Innovation Center, Beijing Technology and Business University, Beijing 100048, China; c School of Agriculture and Biology, Shanghai Jiao Tong University, Shanghai 200240, China; d College of Food Science and Technology, Bohai University; Jinzhou, Liaoning 121013, China

**Keywords:** High-methoxy pectin, Soybean isolate protein, Soyasaponin, Bitterness, Acidic environment

## Abstract

Herein, a soybean protein isolate–soyasaponin (SPI–Ssa) composite system was constructed to mimic a complex, multifaceted soybean product system. This system was stabilized via the addition of high-methoxy pectin (HMP) and low-methoxy pectin (LMP), and the bitter taste of Ssa was modulated. HMP and LMP were compared using sensory evaluation and an electronic tongue assay, revealing that HMP was more effective in suppressing bitterness. Moreover, the interfacial properties and structural characteristics of the SPI–Ssa–HMP composite system were determined at different pH values, and the effect of composite coalescence on the adsorption of Ssa in acidic and neutral environments was investigated. The network structure formed between SPI and HMP was determined by differential scanning isothermal titration calorimetry and Fourier transform infrared spectroscopy. The formation of the network structure between SPI and HMP was more stable at pH 3.0, and the Tp value increased from 145.1 to 155.21 °C.

## Highlights

• Soybean protein isolate–soyasaponin composite system was constructed.
• Bitter taste of Ssa was modulated via the addition of HMP and LMP.
• HMP was more effective in suppressing bitterness.
• Network structure between SPI and HMP was more stable at pH 3.0.
• Force between SPI and HMP was primarily electrostatic interactions.

## Graphical abstract

**Figure:**
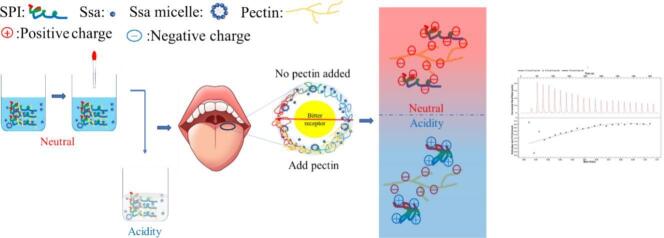

## 1 Introduction

Soy foods are highly nutritious and meet human nutritional requirements. In particular, soybean protein isolate (SPI) provides nine essential amino acids and several functional benefits for food processing and healthy diets (Urade, 2019). It is also an effective source of protein for people allergic to milk proteins. Soyasaponin (Ssa) has high nutritional value and excellent functional properties, such as anti-inflammatory, antiviral, anti-obesity, and free-radical-scavenging effects. During the production process, the soybean hypocotyl is typically removed, and other methods are employed to mask the bitter taste (Bertelsen, Laursen, Knudsen, Møller and Kidmose, 2018a, Bertelsen, Laursen, Knudsen, Møller and Kidmose, 2018b; Tian et al., 2023a, Tian et al., 2023b; Yilmaz & Deviren, 2022). Soy food is not a single component; during its preparation process, it becomes intermingled with Ssa, particularly group A in Ssa, which is a variety of species with the most bitter taste and the main factor affecting the flavor of soy food (Shiraiwa, Harada, & Okubo, 1991). The mechanism underlying the bitter taste is complex, and various compounds contribute to it. Thus, no generalized or established technology for bitter taste inhibition has been developed; however, research into this technology is in progress.

Anti-bittering techniques commonly used in the food industry consist of three main methods: selective separation, enzyme masking (Bertelsen, Laursen, Knudsen, Møller and Kidmose, 2018a, Bertelsen, Laursen, Knudsen, Møller and Kidmose, 2018b), and flavor masking (Tian et al., 2023a, Tian et al., 2023b). Separation mechanisms and enzymatic methods reduce the amount of bitter and bioactive components in the system. Bitterness-masking agents can effectively mitigate or eliminate the perception of bitterness by inhibiting the transmission of bitter taste signals or by disrupting taste integration within the central nervous system. The bitterness intensity can also be masked to a certain extent by interactions between bitterness and other flavors, such as sweet and sour (Ley, 2008). In particular, sweeteners and flavoring additives are often used in this regard.

Citrus fruit cell walls contain a heterogeneous polysaccharide known as pectin, which is primarily composed of galacturonic acid and can undergo partial methoxylation or amidation. Pectin is classified based on its degree of methoxylation, with high-methoxy pectin (HMP) having a degree of esterification (DE) greater than 50 %, and low-methoxy pectin (LMP) having a DE less than 50 % (Ropartz & Ralet, 2020). Currently, pectin extracted from oranges or apples is commonly used in the food industry to solidify or thicken foods and stabilize milk or acidic drinks. As a soluble dietary fiber, it can effectively reduce the risk of heart disease and regulate blood sugar levels (Brouns et al., 2012; Elshahed, Miron, Aprotosoaie, & Farag, 2021; Emran et al., 2022). According to previous studies, polysaccharides are used to modulate bitter flavors in food systems. For example, cyclodextrins are used as debittering agents to mask bitter tastes (Szejtli & Szente, 2005). Moreover, pectin and gum arabic can mask bitter tastes by modulating the anionic nature of pectin to form cohesive complexes with cationic plant proteins. By utilizing the negative charge carried by pectin and the positive charge carried by soy protein, electrostatic interactions form a composite system in which pectin forms a network structure with cationic plant proteins, resulting in better adsorption of the small-molecule Ssa in the system (Agarkova, Kruchinin, Glazunova, & Fedorova, 2019).

In this study, we prepared a multifunctional composite system to simulate the various components of soybean food and explored its bitterness using different Ssa:HMP ratios. The effects of pectin with different degrees of esterification (HMP and LMP) on the bitterness of soybean food were compared and the mechanisms of action and stability of the composite system in acidic and neutral environments were investigated. Moreover, we combined sensory evaluation and electronic tongue experiments to investigate the bitter taste threshold of Ssa. The interfacial characteristics and structural changes in the composite system were examined using Fourier-transform infrared spectroscopy (FTIR), endogenous fluorescence spectroscopy, and differential scanning calorimetry (DSC). The rationale behind the manifestation of bitterness was determined using isothermal titration calorimetry (ITC). Although analyzing the interactions among pectin, SPI, and Ssa from a molecular microscopic perspective and identifying the specific sites of action is outside the scope of this study, the formation of network structures in the composite system can be investigated in future research through molecular simulation. This study offers a theoretical framework for enhancing the taste profile of soybean-based foods, bolstering the scientific and technological comprehension of China's soybean processing sector and consequently amplifying its economic advantages.

## 2 Materials and methods

### 2.1 Materials

Ssa with a purity exceeding 80 % was sourced from Tongze Biotechnology Co., Ltd. (Xi’ an, China). Defatted soy flour was supplied by Shandong Yuwang Industrial and Commercial Co. Ltd. (China). SPI was prepared using an in-laboratory extraction process. Two varieties of commercial pectin derived from citrus and apple sources were procured from Shanghai Yuanye Biotechnology Co. (Shanghai, China). The degrees of esterification of these two pectins were 71 % and 38 %, respectively. Analytically pure KCl, NaH_2_PO_4_·2H_2_O, Na_2_HPO_4_·12H_2_O, HCl, NaOH, KBr, and tartaric acid were acquired from Sinopharm Chemical Reagents Co., Ltd. (Shanghai, China).

### 2.2 Preparation of raw materials

#### 2.2.1 Extraction of SPI

The SPI was extracted according to previously reported methods. Dried, defatted soybean meal was crushed and dispersed in deionized water, and the pH of the dispersible solution was adjusted using 2 M NaOH and HCl. According to the principle of alkali dissolution and acid precipitation, the solution was centrifuged several times (4 °C, 3300 g, 30 min) to obtain SPI precipitates, which were then lyophilized to obtain SPI powder (−40 °C, 20 Pa, 12 h) (Li et al., 2024; T. Wang et al., 2021). Using the Biuret method, the SPI was determined to be comprised of 97.78 % ± 0.005510 % peptides (Lijuan, Zhanmei, Xuelin, & Bo, 2008).

#### 2.2.2 Preparation of SPI-Ssa-HMP/LMP composite system

Lyophilized SPI (1 mg/mL) samples were mixed in different ratios of Ssa, HMP, and LMP (4:1, 2:1, 1:0, 1:1, 1:2, 1:3, 1:4, and 1:5). These mixtures were then dispersed in phosphate buffer solution (pH 7.0, 10 mM). The dispersion process involved gentle magnetic stirring for a duration of 2 h at ambient temperature (25 °C). The samples were stored at 4 °C overnight to allow for thorough hydration. The pH (3.0–7.0) of the ternary composite system was adjusted using 2 M HCl or 2 M NaOH (Wan, Wang, Wang, Yuan and Yang, 2014a, Wan, Wang, Wang, Yuan and Yang, 2014b).

### 2.3 Sensory evaluation

Sensory testing of the SPI-Ssa-HMP/LMP composite system at different pH values involved ten volunteers (healthy, normal sense of smell and taste, aged 18–35 years) who were screened and trained for the experiment. The samples were divided into unified containers and numbered. The tasting intervals of the different samples were set to 30 min. The sensory scores of different flavors (bitterness, astringency, aftertaste-B, and aftertaste-A) of the samples were scored according to the scoring criteria listed in Table S1 (Li et al., 2024). All authors' institutions granted ethical approval to conduct the sensory studies. In the sensory assessment study involving human subjects, the following measures were taken:

1) Protocols were strictly adhered to such that the rights and privacy of all individuals involved in the research were safeguarded.
2) Each participant provided their consent to participate and for their data to be utilized.

### 2.4 Electronic tongue determination

Referring to a previous method with slight modifications, varying proportions of lyophilized SPI-Ssa-HMP/LMP samples were dissolved in ultrapure water (Li et al., 2024; J. Liu et al., 2016). Sample solutions of 100 mL each were filtered through a 0.45 μm microporous water filter membrane, and the resulting filtrates were subsequently used to assess flavor profiles using an electronic tongue device (SA402B, Japan). For comparison, the test samples were evaluated along with a benchmark solution consisting of 30 mM KCl and 0.3 mM tartaric acid.

### 2.5 Turbidity and zeta potential measurements

The turbidity of the composite solutions at different pH values was determined at a wavelength of 600 nm using a UV spectrophotometer (L5, China); an equal volume of phosphate buffer was used as a blank control (C. Liu et al., 2023).

Samples of the individual composite systems were diluted 1:10 with a buffer solution that matched the pH of the sample. The diluted samples were then introduced into the measurement chamber of a zeta potential analyzer (Zetasizer Lab, UK).

### 2.6 Interfacial rheology measurements

The rheological characteristics of the composite mixtures in varying proportions were assessed using a Discovery HR-1 rheometer (TA Instruments, USA). Critical parameters including apparent viscosity, storage modulus (G'), and loss modulus (G') were determined. The specimens were placed on the rheometer's induction plate and covered with a parallel plate measuring 40 mm in diameter. The distance between the plates was fine-tuned to 10 m. A preliminary oscillatory frequency sweep was executed at a strain level of 0.07 % within the linear viscoelastic limit of the material, spanning frequencies from 0.01 to 1 Hz (Rossetti, Yakubov, Stokes, Williamson, & Fuller, 2008).

### 2.7 Endogenous fluorescence spectroscopy

The influence of pH on the intrinsic fluorescence of soy protein isolate (SPI) in the SPI–Ssa–HMP/LMP composite system was examined using a fluorescence spectrophotometer (F-7000, Japan). The composite samples were prepared in phosphate buffer solutions with varying pH levels. The excitation wavelength was fixed at 295 nm and the emission spectrum was recorded between 310 and 500 nm. The excitation and emission bandwidths were adjusted to 2.5 nm.

### 2.8 Differential scanning calorimetry

The temperature range of the differential scanning calorimeter (Q2000, TA, USA) was set to 20–250 °C at a rate of 10 °C/min for samples weighing 8–10 mg. Three measurements were performed in parallel, and the experimental data were processed and analyzed using the TA Universal Analysis 2000 software to obtain the average values.

### 2.9 FTIR spectroscopy

FTIR spectroscopy (JSM-6490LV, Japan) was performed at scanning wavelengths, resolutions, and scan numbers of 4000–400 cm^−1^, 4 cm^−1^, and 32 scans, respectively. The samples were combined with KBr at a ratio of 1:100 before the assay and pressed into pellets. The measurements were obtained after subtracting the blank background.

### 2.10 Isothermal titration calorimetry

Isothermal titration calorimetry (ITC) measurements were conducted using a Nano ITC instrument (TA, USA) at 25 °C. All samples were prepared by dissolving them in phosphate buffer (10 mM, pH 3.5) and degassing. The SPI solution (300 μL, 0.1 mg/mL) was placed into the cuvette, and 50 μL of HMP solution (0.1 mg/mL) was added using a syringe to adjust the pH to 3.5. Baseline balancing was performed after titration began, with the syringe stirred at 250 rpm and dripping at a rate of 2 μL per drop, followed by the injection of the HMP solution into the SPI solution 22 times. To exclude the influence of liquid solutions on the enthalpy change when injected into each other, three control groups were established: Ssa buffer, buffer-SPI, and buffer-buffer (where the former were added dropwise to the latter). According to the enthalpy change results, the first control group exhibited the greatest influence on the experimental results. Launch Nano Analyze software was used to collect and process the experimental data, and the data from the first control group were eliminated. Finally, an independent binding model was used to fit the data, and the reaction thermodynamic parameters of HMP-SPI were obtained (Li et al., 2024; Wan, Wang, Wang, Yuan and Yang, 2014a, Wan, Wang, Wang, Yuan and Yang, 2014b).

### 2.11 Scanning electron microscopy

Appropriate amounts of lyophilized SPI-Ssa-HMP complexes with different pH values were placed on a sample holder and coated with gold. The surface topography of the samples was analyzed using a scanning electron microscope (SEM5000, Chinainstru& Quantumtech (Hefei) Co.,Ltd., China) set to an acceleration voltage of 2.0 kV.

### 2.12 Statistical analysis

Each experiment was conducted in triplicate. The results were reported as mean ± standard deviation (mean ± SD). The resulting data were analyzed using SPSS Statistics 19 (SPSS Inc., USA) and plotted using Origin 2023 (OriginLab Corporation, USA). A one-way analysis of variance (ANOVA) and Duncan's multiple comparison test were used for significance and correction analyses (*p* < 0.05).

## 3 Results and discussion

### 3.1 Sensory evaluation

The sensory evaluation results of the SPI-Ssa-HMP/LMP composite systems with different ratios are listed in Tables S2 and S3. After establishing the mathematical fuzzy matrix, the method of Li (Li et al., 2024) was used to derive the composite scoring results (Table S1).

The composite scores indicate that bitterness decreased as the percentage of HMP in the Ssa:HMP ratio increased, with the lowest bitterness value at an Ssa:HMP ratio of 1:5. The trend of the SPI–Ssa–LMP composite system was similar to that of SPI–Ssa–HMP; however, it had the lowest bitterness value at an Ssa:LMP ratio of 1:4.

The results of the sensory evaluation of the SPI–Ssa–HMP composite system at different pH values are shown in Table S4, and the composite scores are listed in Table 3. The composite scores revealed that bitterness values increased with increasing pH and were the lowest at pH 3.0. The composite scores at pH 4.0 and 5.0 exhibited similar bitterness values, which were higher at pH 7.0.

### 3.2 Electronic tongue analysis

**Fig. 1:**
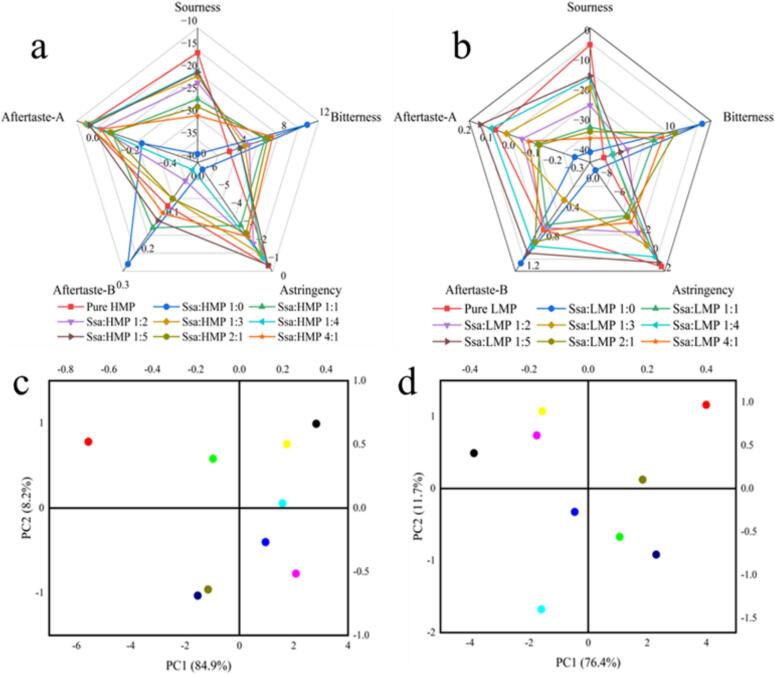
Electronic tongue maps of different scales of SPI-Ssa-HMP (LMP), radargrams of different scales of SPI-Ssa-HMP (a); radargrams of different scales of SPI-Ssa-HMP (b); radargrams of different scales of SPI-Ssa-LMP (c); and PCA analysis maps of different scales of SPI-Ssa-LMP (d).

Composite systems incorporating HMP or LMP were formulated, and the results are shown in Fig. 1. The dominant taste attributes for both systems were bitterness and aftertaste-B, which were predominantly conveyed by Ssa. Principal component analysis (PCA) revealed that the combined contribution rates of bitterness and aftertaste-B were 93.1 % and 88.1 %, respectively. These values exceeding 85 % indicate that the composite systems captured the essence of the overall samples. The PCA plots for the two systems demonstrate a clear separation between the samples, indicating the ability of the tongue to differentiate them.

In the composite system devoid of HMP, bitterness and aftertaste-B levels were higher than those in the other systems with varying proportions of HMP. Additionally, when Ssa was the only component, the bitterness value ranked second, following the HMP-free system. Ultimately, as the HMP content increased, the bitterness and aftertaste-B levels gradually decreased, suggesting that the addition of HMP effectively mitigated the bitterness of the system. The electronic findings of the electronic tongue measurement for the SPI–Ssa–LMP composite system mirrored those of the SPI–Ssa–HMP system. For instance, bitterness and aftertaste were most pronounced in the LMP-free composite system. Furthermore, with increasing LMP content, bitterness levels decreased progressively, aligning with the sensory evaluation outcomes.

In summary, both types of pectin effectively inhibited bitter taste (Huang et al., 2024a, Huang et al., 2024b). The inhibitory effect of HMP on bitterness was stronger than that of LMP; therefore, subsequent experiments were performed using the SPI–Ssa–HMP composite system.

### 3.3 Turbidity and zeta potential analyses

**Fig. 2:**
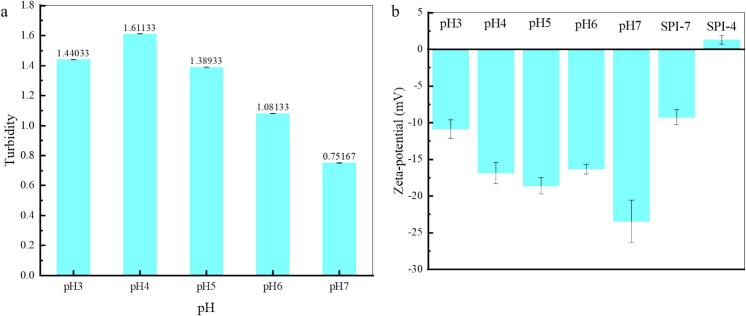
Turbidity and potential plots of the composite system at different pH, a: turbidity; b: zeta-potential (SPI-7, SPI-4: indicate zeta-potentials at pH 7.0 and pH 4.0, respectively).

The cloudiness of a solution reflects the extent of particle clumping, and the zeta potential reveals the electrostatic relationships between proteins and polysaccharides by indicating the surface charge of large molecular particles. Under specific conditions, stronger repulsion between particles leads to a more stable system (Ru, Wang, Lee, Ding and Huang, 2012a, Ru, Wang, Lee, Ding and Huang, 2012b). As shown in Fig. 2a, with an increase in pH in the 3.0–7.0 range, the turbidity exhibits an overall gradual decrease. However, it is the highest at pH 4.0 because SPI and HMP have opposite charges at this pH value. Insoluble complexes were formed because of their strong attraction, and protein alkali–soluble acid precipitation resulted in the formation of protein aggregates. The structural rearrangement of SPI and HMP resulted in a denser and more stable core-shell structure, leading to maximum turbidity (Geng et al., 2023). Based on the zeta potential results at pH 3.0, the opposite charges of SPI and pectin enhance the electrostatic interactions in acidic environments (Wu et al., 2022). At pH 7.0, the incompatible interactions are more likely to dominate. Owing to this incompatibility, either SPI and HMP repelled each other, and no electrostatic interactions occurred between them, or their formed compounds were too small to detect (Han, Shi, Gong and Zhang, 2023a, Han, Shi, Gong and Zhang, 2023b; Hettiarachchi et al., 2016a, Hettiarachchi et al., 2016b). This results in a minimum turbidity value. Moreover, owing to the strong electrostatic repulsion between proteins and polysaccharides with high mutual solubility at pH 7.0, protein–pectin complexes are present as a transparent solution (Han, Shi, Gong and Zhang, 2023a, Han, Shi, Gong and Zhang, 2023b; Ru, Wang, Lee, Ding and Huang, 2012a, Ru, Wang, Lee, Ding and Huang, 2012b; Sun, Ding, Zhang, Lai, & Chen, 2023). According to previous studies, the interactions between proteins and pectin are primarily electrostatic, and hydrogen bonds or hydrophobic interactions are also involved (Wu et al., 2022). With a decrease in pH, turbidity and electrostatic interactions gradually increased, and various noncovalent interactions occurred between the pectin molecules and plant proteins. As the pH decreases below the isoelectric point of proteins, their structures begin to unfold, increasing the flexibility of their side chains. Simultaneously, the free carboxyl groups of pectin form electrostatic bonds with the positively charged protein molecules. In addition, the hydrophobic methoxy or acetyl groups present in pectin can interact with hydrophobic amino acid residues in proteins, leading to the formation of a network structure.

The results revealed that electrostatic attraction was strongest at pH 3.5, which helped form the complex, and higher turbidity was confirmed at this pH (Geng et al., 2023). Free Ssa molecules and micelles were adsorbed in the network structure, with some non-covalently interacting with expanded protein side chains. These interactions trap Ssa in the network structure, effectively reducing bitterness.

### 3.4 Rheological property analysis

**Fig. 3:**
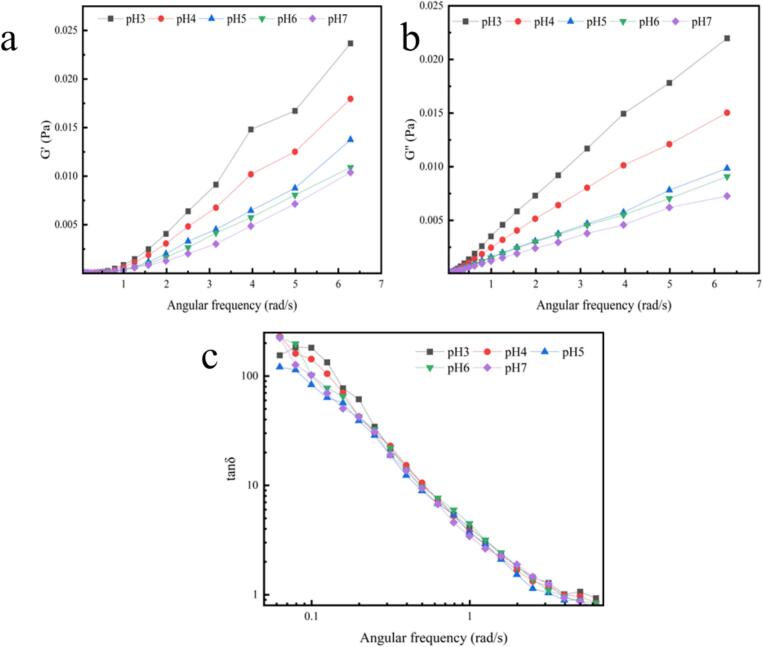
Relationship between (a) storage modulus G' and (b) loss modulus G"and (c)tanδ of SPI-Ssa-HMP condensates prepared at different pH.

According to previous reports, G' reflects the strength of electrostatic interactions between proteins and polysaccharides, with higher G' values indicating stronger electrostatic interactions (Lan, Ohm, Chen, & Rao, 2020a). As shown in Fig. 3, as pH decreases from 7.0 to 3.0 (neutral to acidic environment), G' exhibits an increasing trend. The higher G' values in acidic environments suggest stronger electrostatic interactions between SPI and HMP molecules within the network structure, likely due to protein–protein and protein–polysaccharide interactions (Ru et al., 2012). Since the G" value exceeds G', the condensed layer exhibits strong liquid viscoelastic behavior. Tan δ values decrease moderately with increasing frequency, but do not reach 1 (data not shown). This behavior also confirms the existence of cross-linked networks (Espinosa-Andrews, Sandoval-Castilla, Vázquez-Torres, Vernon-Carter and Lobato-Calleros, 2010a, Espinosa-Andrews, Sandoval-Castilla, Vázquez-Torres, Vernon-Carter and Lobato-Calleros, 2010b; Ling, Huang, He, & Zhou, 2024; Zhang, Li, Sun, Lai, & Chen, 2023).

### 3.5 Endogenous fluorescence spectra of the composite system

**Fig. 4:**
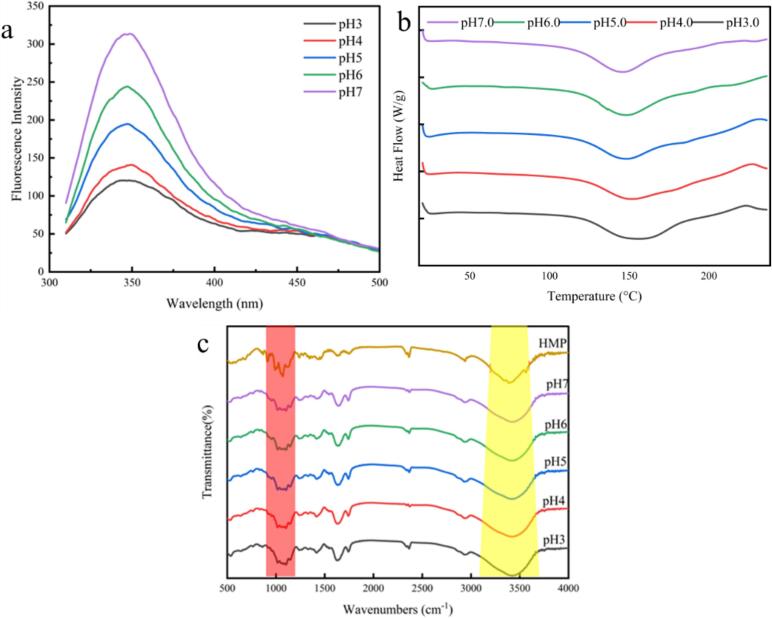
(a) The endogenous fluorescence spectroscopy; (b) Thermal properties of SPI-Ssa-HMP at different pHs; (c) FTIR spectra of SPI-Ssa-HMP at different pHs.

Fig. 4a shows the change in fluorescence intensity with changes in pH. As pH decreases, the fluorescence intensity of the complex also decreases, supporting previous conclusions that a complex is formed. Fig. 4a also shows that λ_max_ exhibits a red shift (312.9–121.4 nm) in the 7.0–3.0 pH range, indicating that pH changed the conformation of the protein in the composite system. The observed effect is attributed to the disruption of the compact structure of the protein and the subsequent exposure of tryptophan and tyrosine residues to a polar milieu. And in an acidic environment, proteins are mostly positively charged after defolding and bind to acidic pectin more through electrostatic interactions. As the number of binding sites between the protein and HMP increased, more HMP molecules encircled the SPI surface, leading to a progressive reduction in SPI's fluorescence intensity of SPI. Analogous findings were reported in studies on the interaction between potato protein and pectin, as well as between β-lactalbumin and λ-carrageenan under varying pH conditions (Han, Shi, Gong and Zhang, 2023a, Han, Shi, Gong and Zhang, 2023b; L. Wang, Yue, Wang, Bai, & Li, 2019).

### 3.6 Thermal performance

DSC is an effective technique for assessing the impact of heat on the physical and chemical characteristics of a substance. In DSC analysis, the temperature at which the most significant peak occurs is typically used to determine the denaturation temperature, which serves as a measure of the thermal stability of a compound (Han et al., 2023). According to the DSC analysis shown in Fig. 4b, the pH of the lyophilized samples of the composite system shifts from neutral to acidic and the heat flow peak shifts to higher temperatures. Moreover, the Tp value increases from 145.1 to 155.21 °C, indicating that the composite cohesive system formed by SPI–HMP via electrostatic interactions is more stable. This can be attributed to the establishment of intermolecular linkages between pectin and proteins, which facilitated the creation of more robust proteoglycan assemblies (X.-x. Wang, Yue, et al., 2019). According to earlier studies, the interaction between these two macromolecules initiates cross-linking and bolsters the architecture of the complex (Xiao et al., 2020).

### 3.7 FTIR analysis

The effect of the pH on the composite system was further investigated using FTIR spectroscopy. Fig. 4c shows that, based on the previous conclusions, the electrostatic interactions between the composite systems become more pronounced as the pH decreases. According to the FTIR results, characteristic peaks are observed at 1745 and 900–1250 cm^−1^, indicating that SPI and HMP were bound by electrostatic interactions. Owing to the electrostatic interaction between the amino group (–NH ^3+^) of SPI and the carboxyl group (–COO–) of HMP, the peaks of amides I (1600–1700 cm^−1^), II (1450–1550 cm^−1^), and III (1200–1450 cm^−1^) shift to higher wavenumbers at all pH values. Similar results were obtained for complex systems of egg white protein-xanthan gum and senna-chitosan gum (Souza & Garcia-Rojas, 2017; You, Liu, & Zhao, 2018). Furthermore, an extensive peak ranging from 3199 to 3596 cm^−1^ was assigned to the O—H stretching vibration, signifying alterations in either inter- or intramolecular hydrogen bonding. Based on the hydrogen bond interactions between SPI and Ssa, the hydrophilic carboxyl, hydroxyl, and amide groups from both proteins and pectin can form hydrogen bonds. Coupled with the fact that the composite system absorbs Ssa, the development of a network structure, leading to a decrease in free Ssa in the bulk phase, contributes to the reduction in bitter taste (Wu et al., 2022).

### 3.8 ITC analysis

**Fig. 5:**
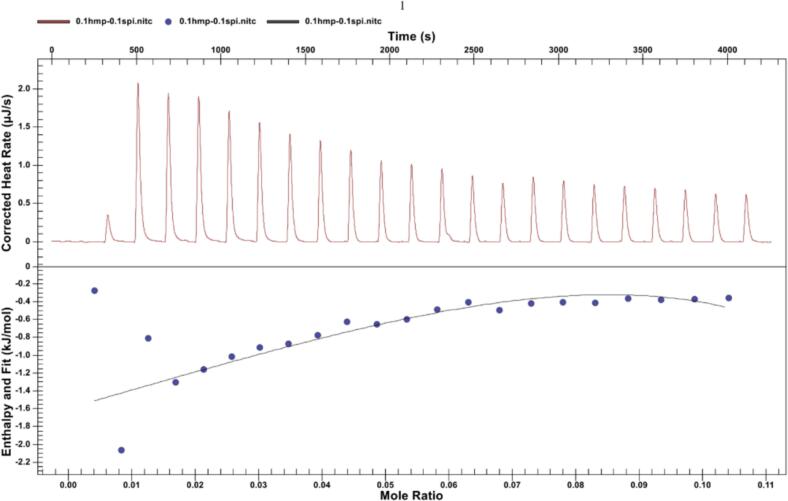
Thermal analysis plots (upper panel) and binding isotherms (lower panel) of SPI titrated against a dispersion of HMP in phosphate buffer (pH 3.5) at 25 °C.

To understand the mechanism of network structure formation in the composite system, ITC was used to determine the thermodynamic parameters of complexation. Typical heat flow versus time curves are shown in Fig. 5. The injection exhibits an exothermic reaction, indicating nonspecific electrostatic neutralization between the opposing charges of SPI and HMP and implying an enthalpic drive from the complex condensation process (Girard, Turgeon, & Gauthier, 2003; Schmitt et al., 2005). Comparable exothermic patterns have been documented in systems involving ovalbumin–chitosan and soy protein–gum arabic interactions (Dong et al., 2015; Xiong et al., 2016). A suite of thermodynamic parameters was derived by aligning the titration curve with an independent binding model. The binding constant (Ka) of SPI and HMP is 1 × 10^3^ M^−1^, indicating a weak interaction. The unfavorable Gibbs free energy change (ΔG of −17.12 kJ/mol), along with the negative enthalpy (ΔH) and entropy (ΔS) values of −28.98 and − 30.00 kJ/mol respectively, signify the favorable and spontaneous complexation between proteins and pectin. This process is predominantly enthalpy driven because of electrostatic interactions and hydrogen bonding, with the entropy change due to counterion release being a secondary factor (Lan, Ohm, Chen, & Rao, 2020b; Ross & Subramanian, 1981). The combination of soybean protein and gum arabic primarily exhibits electrostatic interactions.

### 3.9 Microstructure analysis

**Fig. 6:**
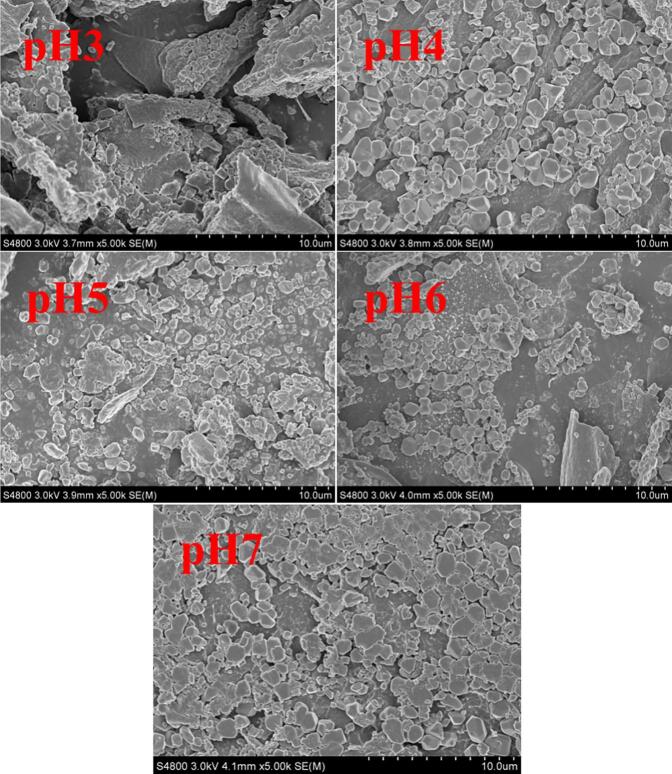
SEM micrographs of the composite system at different pH (×5000).

Microstructural analyses of SPI and SPI–Ssa revealed lamellar and spherical aggregate shapes, respectively, which are similar to those in previous studies (Geng et al., 2023). As shown in Fig. 6, the microstructure of the composite system is dense in the acidic environment, and the number of globular aggregates present on the surface gradually decreases. Combined with these results, the endogenous fluorescence spectroscopy, FTIR, and DSC results revealed that electrostatic interactions between SPI and HMP formed a network structure (Sun et al., 2023). The stability of the network structure was stronger at pH 3.0, facilitating the encapsulation of Ssa and reducing its bitter taste, which is consistent with the results of the organoleptic evaluation.

## 4 Conclusions

This investigation elucidates that the strategic integration of pectin polysaccharides with soy protein-saponin complexes effectively mitigates undesirable bitterness through tailored molecular entrapment mechanisms. Sensory profiling coupled with instrumental analysis established optimal bitterness suppression at distinct biopolymer ratios (1:5 for HMP and 1:4 for LMP), with HMP demonstrating superior masking capabilities attributed to its structural specificity. pH-dependent interfacial reorganization studies revealed enhanced colloidal stability under acidic conditions (pH 3.0), where intensified electrostatic associations between SPI and HMP fostered robust network formation. Thermal denaturation analysis confirmed improved structural integrity through increased transition temperatures (Tp: 145.1–155.21 °C), while thermodynamic parameters (ΔH = −28.98 kJ/mol; ΔS = −30.00 kJ/mol) validated the spontaneous binding process driven by charge-mediated interactions. These findings advance the rational design of plant-based food matrices by demonstrating how biopolymer selection and environmental modulation synergistically control bioactive compound bioavailability and sensory perception.

## CRediT authorship contribution statement

**Lijie Zhu:** Writing – original draft, Data curation, Conceptualization. **Yingyan Li:** Writing – original draft, Methodology, Investigation. **He Li:** Formal analysis. **Dayun Zhao:** Formal analysis. **Xinqi Liu:** Data curation. **Qian Shen:** Software. **Qi Zhang:** Writing – review & editing. **Qingyun Lv:** Writing – review & editing. **Xiuying Liu:** Writing – review & editing, Conceptualization. **Wenping Ding:** Writing – review & editing, Conceptualization.

## Declaration of competing interest

The authors declare that they have no known competing financial interests or personal relationships that could have appeared to influence the work reported in this paper.

## Data Availability

No data was used for the research described in the article.

## Appendix A Supplementary data

Supplementary material Image 1
