# Supplementary material for: Mechanistic investigation of a soybean protein isolate–soyasaponin–pectin composite system on soyasaponin bitterness in an acidic environment
Source: Food Chem X. 2025 May 1;27:102500. doi: 10.1016/j.fochx.2025.102500 (PMC12124663; doi:10.1016/j.fochx.2025.102500)
Supplement: Supplementary file 1 — Supplementary material [file mmc1.docx]

Table S 1 Sensory evaluation criteria of Ssa-SPI composite system with different concentrations

| Indicators (scores) | Grading standard (score) | | |
| --- | --- | --- | --- |
|  | Satisfactory | Medium | Not satisfactory |
| Bitterness (40) | Non-bitter (27-40) | A little bitter (14-26) | Bitter heavy (1-13) |
| Astringency (30) | Non- astringent (21-30) | A little astringent (11-20) | astringent heavy (1-10) |
| Aftertaste-B (15) | ＜1s(11-15) | 2-5s (6-10) | ＞5s(1-5) |
| Aftertaste-A (15) | ＜1s(11-15) | 2-5s (6-10) | ＞5s(1-5) |

Table S 2 Sensory evaluation results of SPI-Ssa-HMP ternary composite system with different ratios

| Ssa:HMP | Bitterness（40 points） | | | Astringency（30 points） | | | Aftertaste-B（15 points） | | | Aftertaste-A（15 points） | | |
| --- | --- | --- | --- | --- | --- | --- | --- | --- | --- | --- | --- | --- |
|  | Satisfactory  27-40 | Medium  14-26 | Not satisfactory  1-13 | Satisfactory  21-30 | Medium  11-20 | Not satisfactory  1-10 | Satisfactory  11-15 | Medium  6-10 | Not satisfactory  1-5 | Satisfactory  11-15 | Medium  6-10 | Not satisfactory  1-5 |
| Pure HMP | 10 | 0 | 0 | 9 | 1 | 9 | 1 | 0 | 1 | 9 | 1 | 0 |
| 1:0 | 0 | 1 | 9 | 8 | 2 | 0 | 2 | 8 | 3 | 9 | 1 | 0 |
| 1:1 | 2 | 2 | 6 | 9 | 1 | 0 | 3 | 2 | 5 | 9 | 1 | 0 |
| 1:2 | 2 | 2 | 6 | 9 | 1 | 0 | 8 | 2 | 0 | 9 | 1 | 0 |
| 1:3 | 2 | 3 | 5 | 9 | 1 | 0 | 9 | 1 | 0 | 8 | 2 | 0 |
| 1:4 | 9 | 1 | 0 | 9 | 1 | 0 | 9 | 1 | 0 | 8 | 2 | 0 |
| 1:5 | 1 | 0 | 0 | 9 | 1 | 0 | 9 | 1 | 0 | 9 | 1 | 0 |
| 2:1 | 1 | 2 | 7 | 9 | 1 | 0 | 7 | 3 | 0 | 8 | 2 | 0 |
| 4:1 | 0 | 2 | 8 | 9 | 1 | 0 | 6 | 4 | 0 | 7 | 2 | 1 |

Table S 3 Sensory evaluation results of SPI-Ssa-LMP ternary composite system with different ratios

| Ssa:LMP | Bitterness（40 points） | | | Astringency（30 points） | | | Aftertaste-B（15 points） | | | Aftertaste-A（15 points） | | |
| --- | --- | --- | --- | --- | --- | --- | --- | --- | --- | --- | --- | --- |
|  | Satisfactory  27-40 | Medium  14-26 | Not satisfactory  1-13 | Satisfactory  21-30 | Medium  11-20 | Not satisfactory  1-10 | Satisfactory  11-15 | Medium  6-10 | Not satisfactory  1-5 | Satisfactory  11-15 | Medium  6-10 | Not satisfactory  1-5 |
| Pure LMP | 8 | 2 | 0 | 7 | 1 | 2 | 8 | 1 | 1 | 9 | 0 | 1 |
| 1:0 | 0 | 0 | 10 | 9 | 1 | 0 | 0 | 1 | 9 | 9 | 1 | 0 |
| 1:1 | 0 | 2 | 8 | 9 | 1 | 0 | 8 | 1 | 1 | 9 | 1 | 0 |
| 1:2 | 3 | 2 | 5 | 9 | 1 | 0 | 8 | 2 | 0 | 9 | 1 | 0 |
| 1:3 | 4 | 2 | 4 | 7 | 3 | 0 | 8 | 2 | 0 | 8 | 1 | 1 |
| 1:4 | 4 | 3 | 3 | 8 | 2 | 0 | 8 | 2 | 0 | 8 | 0 | 2 |
| 1:5 | 4 | 1 | 5 | 7 | 1 | 2 | 8 | 1 | 1 | 7 | 0 | 3 |
| 2:1 | 0 | 1 | 9 | 9 | 1 | 0 | 8 | 1 | 1 | 9 | 1 | 0 |
| 4:1 | 0 | 1 | 9 | 9 | 1 | 0 | 8 | 2 | 0 | 9 | 1 | 0 |

Table S 4 Sensory evaluation results of SPI-Ssa-HMP composite system with different pH

| pH | Bitterness（40 points） | | | Astringency（30 points） | | | Aftertaste-B（15 points） | | | Aftertaste-A（15 points） | | |
| --- | --- | --- | --- | --- | --- | --- | --- | --- | --- | --- | --- | --- |
|  | Satisfactory  27-40 | Medium  14-26 | Not satisfactory  1-13 | Satisfactory  21-30 | Medium  11-20 | Not satisfactory  1-10 | Satisfactory  11-15 | Medium  6-10 | Not satisfactory  1-5 | Satisfactory  11-15 | Medium  6-10 | Not satisfactory  1-5 |
| 3.0 | 9 | 1 | 0 | 10 | 0 | 0 | 10 | 0 | 0 | 10 | 0 | 0 |
| 4.0 | 8 | 2 | 0 | 9 | 1 | 0 | 9 | 1 | 0 | 8 | 2 | 0 |
| 5.0 | 8 | 2 | 0 | 8 | 2 | 0 | 7 | 3 | 0 | 8 | 2 | 0 |
| 6.0 | 7 | 1 | 2 | 8 | 2 | 0 | 8 | 2 | 0 | 8 | 1 | 1 |
| 7.0 | 5 | 1 | 4 | 6 | 1 | 3 | 8 | 1 | 1 | 7 | 1 | 2 |

Table S5 Sensory evaluation composite scores of SPI-Ssa-HMP composite system with different pH

| pH | Yi Evaluation result sets | Comprehensive score |
| --- | --- | --- |
| 3.0 | Y1={0.96, 0.04, 0} | 89.2 |
| 4.0 | Y2={0.845, 0.155, 0} | 86.9 |
| 5.0 | Y3={0.785, 0.215, 0} | 85.7 |
| 6.0 | Y4={0.733, 0.13, 0.14} | 81.8 |
| 7.0 | Y5={0.605, 0.1, 0.295} | 76.2 |
